# Case report and review: Angiosarcoma with thrombocytopenia after total hip arthroplasty

**DOI:** 10.3389/fsurg.2023.1212491

**Published:** 2023-07-31

**Authors:** Noora Skants, Mikko Rönty, Olli Komulainen, Miikka Keski-Keturi, Kaisa Huotari, Maria Alander-Pekkarinen, Marjut Sihvo, Minna Laitinen, Rita Linko

**Affiliations:** ^1^ Perioperative and Intensive Care, Peijas Hospital, Helsinki University Hospital and University of Helsinki, Vantaa, Finland; ^2^ Department of Pathology, HUSLAB, Hospital District of Helsinki and Uusimaa, Diagnostic Center and University of Helsinki, Helsinki, Finland; ^3^ Musculoskeletal and Plastic Surgery, Arthroplasty Center, Peijas Hospital, Helsinki University Hospital and University of Helsinki, Vantaa, Finland; ^4^ Emergency Medicine and Services, Helsinki University Hospital and University of Helsinki, Helsinki, Finland; ^5^ Department of Infectious Diseases, Helsinki University Hospital and University of Helsinki, Helsinki, Finland; ^6^ Internal Medicine and Rehabilitation, Helsinki University Hospital and University of Helsinki, Helsinki, Finland; ^7^ Musculoskeletal and Plastic Surgery, Bridge Hospital, Helsinki University Hospital and University of Helsinki, Helsinki, Finland

**Keywords:** total hip arthroplasty, endoprosthesis, pseudotumor, angiosarcoma, thrombocytopenia

## Abstract

Total hip arthroplasty (THA) is a common treatment for osteoarthritis and is also performed for other conditions, such as secondary arthritis due to developmental dysplasia of the hip. Various THA types may be complicated by osteolysis and an inflammatory pseudotumor due to an adverse reaction to metal debris. Rarely, THA has been associated with malignant tumors, but their causality remains unclear. In this case report, we describe a female patient with developmental dysplasia of the hip. She had undergone left metal-on-polyethylene THA, acetabular revision of the THA, and left total knee arthroplasty. In addition, she had a history of dyslipidemia and telangiectasia of the eyes, anemia, hiatal hernia, and pleuritis. A THA-associated mass (suspected to be a pseudotumor) had been detected during a previous hospital admission due to pleuritis. She was hospitalized due to swelling in her left lower limb, fatigue, and bruises. A clinical examination revealed anemia, thrombocytopenia, and growth of the suspected pseudotumor. Within 6 weeks, she presented with bleeding of the oral mucosa, hemoptysis, melena, severe thrombocytopenia that did not respond to treatment, elevated D-dimer and C-reactive protein levels, severe pain, increased osteolysis, and fractures around the THA. Infection or malignancy was suspected, but two trocar biopsies suggested an inflammatory pseudotumor. Since her anemia and thrombocytopenia were considered to have been caused by an inflammatory process within the suspected pseudotumor, her suspected pseudotumor and all THA components were surgically removed. However, she developed severe alveolar hemorrhaging and hypoxia and died 2 weeks after her surgery. Histopathological analysis of her surgical and autopsy samples revealed highly malignant angiosarcoma. Although individual cases of malignancies associated with THA have been reported, the literature lacks a clear association between THA and increased cancer risk. Most pseudotumors are non-malignant. The patient's case presented in this report exemplifies the challenges to the differential diagnosis of a THA-associated pseudotumor and rare angiosarcoma. Atypically rapid tumor growth, severe osteolysis, and deterioration in the general wellbeing suggest a malignant disease.

## 1. Introduction

Total hip arthroplasty (THA) is a common treatment for end-stage osteoarthritis and is also performed for other conditions, such as secondary arthrosis due to developmental dysplasia of the hip. Periprosthetic osteolysis and aseptic loosening of the endoprosthesis (40%), dislocation (14%), and infections (11%) are among the most common reasons for THA revision surgery (1).

Occasionally, osteolysis presents with an inflammatory soft-tissue mass called a “pseudotumor.” A pseudotumor is an inflammatory adverse reaction to metal debris (ARMD) that is typically associated with metal-on-metal (MoM) weight-bearing prostheses. It can emerge within 5 years after primary arthroplasty (2, 3). In addition to MoM THA, a pseudotumor may also develop with other weight-bearing endoprostheses—that is, metal-on-polyethylene (MoP) and ceramic-on-polyethylene (CoP) THA—or even hemiarthroplasty of the hip (2, 4–6).

In MoM surfacing arthroplasties, the wear of cobalt and chromium (Cr) particles causes necrotic and inflammatory changes, possibly through a cytotoxic response and delayed hypersensitivity reactions (7). THA-associated ARMD also includes vasculitis-like lesions, such as aseptic lymphocytic vasculitis-associated lesions (ALVALs) (8). Metal particles can spread locally as well as to the lymph nodes, liver, and spleen (9, 10). Even after prolonged metal exposure, an autopsy revealed no end organ damage (10). In a CoP THA-associated pseudotumor, polyethylene wear and histiocytes containing titanium particles have been reported (11). Meanwhile, in an MoP THA-associated pseudotumor, polyethylene particles surrounded by macrophages, without metal debris, have been detected (12). Polyethylene has been suggested to be carcinogenic in animals (13), but evidence of this impact among humans is limited.

Although malignant soft-tissue tumors have been detected around various orthopedic implants, THA had not been associated with an increased cancer or mortality incidence rate in large cohort studies (14, 15). To evaluate the risk of ARMD-associated cancer, studies have integrated data from total joint registries, cancer registries, and hospital records. The Finnish Arthroplasty Registry reveals that the cancer incidence rate of arthroplasty patients did not increase compared with that of the general population (14, 16–18). However, the Swedish Arthroplasty Register shows that in arthroplasty patients, the risk of prostate cancer, melanoma, and multiple myeloma increased in a long-term follow-up (18). In the United Kingdom, the incidence rate of new cancers following THA was lower compared with the general population matched for age and sex (19). In a registry study conducted by Kane et al., it was found that arthroplasty patients had an increased risk of myeloma and monoclonal gammopathy of undetermined significance, although the absolute risks associated with these conditions were low (20).

In a long-term follow-up involving Finnish patients that examined the risk of sarcomas (21), no sarcomas were detected at the sites of THA. In a later cohort study, seven soft-tissue sarcomas were found, and sarcoma risk was higher for the MoM cohort than the non-MoM cohort (16). An angiosarcoma (AS) registry study (22) and cohort studies involving AS patients (23, 24) found no associations between AS and orthopedic implants.

Thus, THA is associated with ARMD, inflammatory pseudotumor, and ALVAL. Although individual cases of malignancies, including sarcomas, have been associated with THA, evidence of their causality or a higher cancer incidence rate among arthroplasty patients is lacking compared with the general population. In addition to our brief literature review, in this case report, we describe a novel case of MoP THA associated with a mass (a suspected pseudotumor that was later confirmed to be an AS) and severe thrombocytopenia that suggested malignancy.

## 2. Case description

This case report was published with the informed consent of the legal representatives of the described patient. Table 1 presents a timeline and summary of her case.

**Table 1:** Timeline of findings, diagnoses, and surgery for the THA-associated pseudotumor and thrombocytopenia case.

Age	Timeline	Findings, diagnoses, and surgery
Birth		Developmental dysplasia of the hip
40		Left THA due to secondary arthrosis
50		Acetabular revision of the left THA
68		Left TKA
71		Routine control for cholesterol; anemia and hiatal hernia detected
72		Cough, dyspnea, CT: nodules at the right lung
73		Abdominal pain (unable to eat), dyspnea, cough x-ray and CT: empyema, thickening of mediastinal pleura
74	Day 0	GP visit for left-limb edema, fatigue, and bruising Lab: anemia, thrombocytopenia. MDS?
	Day 7	Hospital admission: pain, left-limb edema, palpable abdominal tumor, anemia, and thrombocytopenia CT: small peripheral-opacity nodules in the lungs; PE cannot be excluded; left THA-associated pseudotumor, size 6 cm × 7.5 cm × 9 cm; pubic and trochanter fractures; PE? DVT? Bleeding? MDS? ITP? Cold AIHA? DIC? Malignancy?
	Day 18	Pain when walking; no signs of bleeding Hematologists: a peripheral mechanism for thrombocytopenia
	Day 31	No improvement after dexamethasone or IVIG (for ITP) MARS-MRI: several pseudotumors on iliopsoas (7.1 cm × 6.6 cm × 8 cm), posteromedial and caudally to the acetabulum (4.8 cm × 4.8 cm × 8.8 cm), and the fractured trochanter major (4.1 cm × 2.1 cm × 6.4 cm)
	Day 42	Hemoptysis, alveolar hemorrhage
	Day 44	Bruises, hemoptysis, alveolar hemorrhage, moderate hypoxia Hematologists: inflammatory process at the THA pseudotumor with possible bleeding, vasculitis, or microangiopathy
	Day 45	Confusion/incoherence Stomach and gingival bleeding, hematoma of the left ankle Moderate to severe hypoxia due to alveolar hemorrhage
	Day 53	Melena and hemoptysis
	Day 57	Excision arthroplasty surgery: pseudotumor excision, removal of all components of left THA (Girdlestone situation)
	Day 58	Poor cognition, hypoxia, hypotension, and oliguria
	Day 60	Somnolent, hypoxia/dyspnea, and moderate kidney insufficiency
	Day 61	Hypoxia/dyspnea and atrial fibrillation paroxysm
	Day 66	Hypoxia and agitation
	Day 67	Histopathologic diagnosis of surgical resection samples: AS; palliative care
	Day 68	Severe hypoxia and death

AIHA, autoimmune hemolytic anemia; AS, angiosarcoma; CT, computed tomography; DIC, disseminated intravascular coagulation; DVT, deep vein thrombosis; GP, general practitioner; ITP, idiopathic thrombocytopenic purpura; IVIG, intravenous immunoglobulin; MARS-MRI, metal artifact–reducing sequence magnetic resonance imaging; MDS, myelodysplastic syndrome; PE, pulmonary embolism; THA, total hip arthroplasty; TKA, total knee arthroplasty.

### 2.1. Medical and surgical history

This case involved a 74-year-old female patient who had never smoked (weight: 56 kg; height: 157 cm). Her medical history comprised bilateral developmental dysplasia of the hip, parafoveal telangiectasia of the eyes, and dyslipidemia. The only medication she regularly used was atorvastatin.

**Figure 1:**
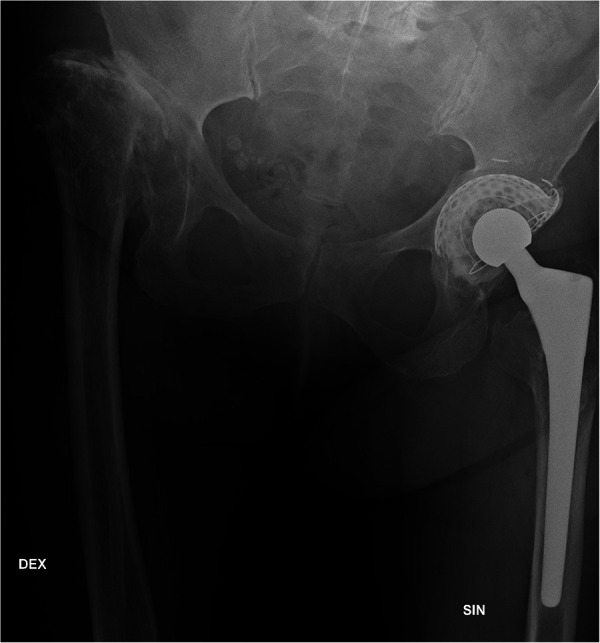
A pelvic x-ray when the patient was 66 years old presents the dislocation of the right hip and the left total hip arthroplasty after acetabular revision. The acetabular cup is augmented with cement and titanium mesh.

The patient had left THA (MoP; Bi-metric™, Biomet Inc, Warsaw, IN) complicated by peripheral deep vein thrombosis (DVT) when she was approximately 40 years old and had undergone revision of the polyethylene acetabular cup (augmented with cement and titanium mesh; Link Orthopaedics UK Ltd., Edinburgh, UK) at 50 years old. She had also undergone left total knee arthroplasty (Nexgen® CR-Flex; Zimmer Biomet, Warsaw, IN) due to arthrosis when she was 68 years old. She had a dislocated right hip (Figure 1) and used crutches to walk.

At the age of 71 years, during a routine check-up with her primary care general practitioner (GP) for dyslipidemia, laboratory tests showed microcytic anemia and iron deficiency. Chest x-rays and gastroscopy showed a large hiatal hernia, and a colonoscopy revealed a small erosion at the flexura hepatica. The *Helicobacter* and carcinoembryonic antigen (CEA) tests of the patient came back negative. The patient had used acetylsalicylic acid (ASA) to prevent cardiovascular events. Her ASA use was discontinued, and her anemia was corrected with a red blood cell (RBC) transfusion and an intravenous (IV) iron infusion.

### 2.2. Examinations for cough, dyspnea, and abdominal discomfort

When the patient was 72 years old, she was examined for cough and dyspnea. Due to a slightly elevated D-dimer (a fibrin degradation product) level, a chest computed tomography (CT) scan with angiography was performed to exclude pulmonary embolism (PE). A non-specific nodule (7 mm in diameter, possibly a lymph node) was found in the lower lobe of her right lung.

**Figure 2:**
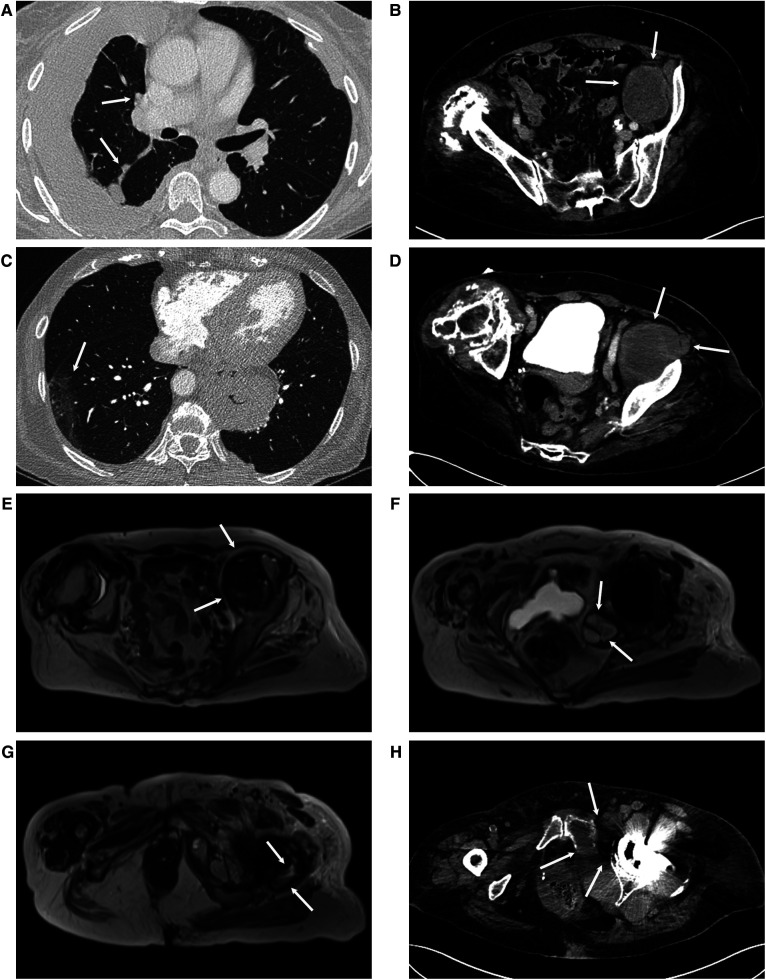
(**A**) A chest CT scan when the patient was 71 years old showed pleural fluid, suggesting empyema. On the right side, the pleura is thickened at the mediastinum and the interlobular septa, suggesting malignancy (arrows). (**B**) At the same time, an abdominal CT scan showed a mass, which was presumed to be a total hip arthroplasty (THA)-associated pseudotumor (arrows). (**C**) When the patient was 74 years old, upon hospital admission, a body CT scan showed ground-glass opacity areas in the lungs (arrow) and (**D**) enlargement of the THA-associated pelvic mass (size: 6 cm × 7.5 cm × 9 cm; arrows). Metal artifact–reducing sequence magnetic resonance imaging (MARS-MRI) showed an enlarged THA-associated mass in multiple locations: (**E**) the iliopsoas region (7.1 cm × 6.6 cm × 8.9 cm, arrows), (**F**) posteromedial to the acetabulum (4.7 cm × 4.8 cm × 8.8 cm, arrows), and (**G**) near the trochanter major (4.1 cm × 2.1 cm × 6.4 cm, arrows). (**H**) A preoperative pelvic CT scan showed remarkable osteolysis of the pubic and acetabular regions (arrows).

At the age of 73 years, the patient was admitted to the Helsinki University Hospital (HUH; Jorvi Hospital, Espoo, Finland) emergency department (ER) after experiencing a couple of weeks of dyspnea, coughing, abdominal discomfort, and difficulties eating normally. Chest x-rays showed pleural fluid on the right side, suggesting pleuritis, empyema, or malignancy. The CT scan revealed pleural fluid resembling empyema, thickening of the mediastinal pleura, and some nodules on the right side of the interlobular pleura (Figure 2A). These results suggested malignancy or tuberculosis (TB), but neither malignant cells, TB, nor other bacteria were found in the pleural fluid samples of the patient. An abdominal CT scan detected a mass (initially suspected to be a pseudotumor; size: 4.7 cm × 6.3 cm × 7.5 cm; Figure 2B) associated with her left THA. A thoracoscopy for pleural biopsies was scheduled. Following a prolonged course of antibiotic treatment, her condition and infection markers improved, and the planned thoracoscopy was canceled based on an oncology consultation.

### 2.3. Hospital admission

When the patient was 74 years old, she visited her GP due to swelling of the left lower limb, fatigue, and bruising (Table 1, Timeline Day 0). Her blood cell count showed remarkable anemia [hemoglobin (Hb): 8.8 g/dl] and thrombocytopenia [platelet (PLT) count: 25 × 10^9^/L]. After a consultation with the HUH ER and hematologists, more laboratory tests, a whole-body CT scan, and a bone marrow biopsy were scheduled. The hypothesis of the hematologists included myelodysplastic syndrome (MDS).

A week after she visited her GP (before her CT scan and bone marrow biopsy), the patient was admitted to the HUH ER due to pain, worsening edema, and a bruise on her left lower limb. A clinical examination revealed a palpable mass in her abdomen and pain when flexing her left hip. The patient was afebrile and did not have a history of weight loss. She presented with worsening anemia (Hb: 8.0 g/dl), thrombocytopenia (PLT: 17 × 10^9^/L), and elevated infection markers [white blood cells (WBC): 15.0 × 10^9^/L; C-reactive protein (CRP): 39 mg/L]. Her blood electrolytes, sugar level, liver and kidney function, clotting time, vitamin B12, and folic acid tests were normal, but the ferritin level was elevated. Blood and urine samples were collected for bacterial cultures. A thromboembolic event was suspected, and the patient was started on tinzaparin.

A body CT (including pulmonary angiography) scan showed small areas of ground-glass opacity, predominantly on the right side of the patient (Figure 2C). PE was not evident but could not be definitely excluded. Her abdominal CT scan showed growth of the suspected pseudotumor (to 6 cm × 7.5 cm × 9 cm, Figure 2D) and fractures of the pubic bone and trochanter major. The spleen appeared normal in size. A day later, a lower-limb ultrasound (US) was performed, which ruled out the presence of DVT. Due to a further decrease in the patient's Hb and PLT levels and the need for RBC and PLT transfusions, treatment with tinzaparin was discontinued. The results of her blood and urine bacterial cultures and *Helicobacter* tests were negative. MDS, malignancy, or bleeding (e.g., hematoma of the psoas region) was suspected.

A bone marrow biopsy was performed, and its preliminary results showed negative for MDS, leukemia, lymphoma, and other malignancies. An abdominal CT scan with angiography was performed again, and no signs of bleeding were found. The radiology report indicated that her suspected pseudotumor was large, but there was no significant change in size compared with the scan taken 3 days earlier. The arthroplasty surgeon from the HUH Arthroplasty Center (HUS Peijas Hospital, Vantaa, Finland) was contacted for consultation. Metal artifact-reducing sequence magnetic resonance imaging (MARS-MRI) was recommended but was not immediately available.

A full blood count indicated reticulocytosis (150–310; normal range: 30–108 × 10^9^/L), low RBC fragments (0.8%–1.1%), and elevated WBC with neutrophilia but normal-to-borderline-low lymphocytes. The patient's hemolysis markers (e.g., haptoglobin and bilirubin) and clotting times were normal. Her plasma protein fractions were normal except for borderline–low albumin levels. Lactate dehydrogenase (450–560 U/L; normal range: 155–255 U/L) and D-dimer (30–38 mg/L; normal range: <0.5 mg/ml) were elevated. A Coombs C3d test was positive. An iron panel showed mild iron deficiency and elevated erythropoietin (EPO) levels. Iron was administered intravenously to correct her iron deficiency. Thyroid hormone tests were normal. Paroxysmal nocturnal hemoglobinuria tests were negative. Based on her laboratory results, autoimmune hemolytic anemia (AIHA), cold AIHA, idiopathic thrombocytopenic purpura (ITP), AIHA combined with ITP, and chronic disseminated intravascular coagulation (DIC) were considered possible. A cold hemagglutinin test was negative. Given her possible ITP, the patient received 40 mg of dexamethasone daily for 4 days and IV immunoglobulin (IVIG; 1 mg/kg per day) twice, without a significant response. Because of her poor response to PLT transfusions, hematologists suggested a peripheral mechanism contributing to thrombocytopenia (e.g., bleeding, vasculitis, or another inflammatory reaction).

Further tests were performed, which resulted negative for hepatitis, parvovirus, Epstein–Barr virus, and human immunodeficiency virus. Among the blood coagulation factors, factors VIII and IX, as well as the active von Willebrandt factor (vWF) and vWF antigen, were slightly above the normal ranges. However, factor FVXIII was slightly below the normal range. These findings suggest a reactive response to possible bleeding. The results of the final bone marrow biopsy of the patient showed normal cell lines, normal granulopoiesis and erythropoiesis, and no signs of MDS, leukemia, lymphoma, or other malignancies. A gene test for BCR-ABL—which can diagnose certain hematologic malignancies—was negative. Her interleukin 2 receptor levels were not as high as expected in hemophagocytic lymphohistiocytosis. Her antiphospholipid antibodies were negative.

MARS-MRI of her left THA and suspected pseudotumor showed significant growth of the mass in multiple locations, namely, the iliopsoas region (7.1 cm × 6.6 cm × 8.9 cm, Figure 2E), posteromedial to the acetabulum (4.7 cm × 4.8 cm × 8.8 cm, Figure 2F), and near the fractured trochanter major (4.1 cm × 2.1 cm × 6.4 cm, Figure 2G). The blood metal ion levels of the patient were not increased [cobalt (Co) level was below 0.9 µg/L, and Cr level was below 0.6 µg/L]. An arthroplasty surgeon was contacted for consultation. Since her diagnosis remained unclear and the rapid growth of the suspected pseudotumor in association with MoP THA was very unusual, US-guided 1 cm trocar biopsies with histopathological diagnosis, bacterial samples, and a new pelvic CT scan were requested before a possible surgery.

In the following 5 weeks after the patient's hospital admission, her condition slowly deteriorated. She suffered from hemoptysis, bleeding from oral mucous membranes and gingiva, and melena. Her oxygen saturation (Sat) values decreased to 88%–92%, and her consciousness became impaired. She had tachycardia [pulse (p.): 100] and minor changes in her electrocardiogram (ECG; T-wave inversion in lead III). A catheter was inserted to treat her urinary retention. Her head CT scan showed white matter degeneration that was normal for her age. A chest CT scan showed a wide, diffuse alveolar hemorrhage, and the related radiology report recommended tests for vasculitis. A pelvic CT scan (Figure 2H) showed wide osteolysis and several fractures around the acetabular components, and the related radiology report suggested osteomyelitis or a THA-associated infection. Tests for vasculitis [e.g., anti-neutrophil cytoplasmic antibody (ANCA) and myeloperoxidase] were negative. Because of her poor response to PLT transfusions, the patient was tested for anti-human leukocyte antigen (HLA) class I antibodies. She developed a strong immune response. HLA-typed platelets were needed, which significantly delayed the trocar biopsies. WBC (23 × 10^9^/L) and CRP (145 mg/L) levels were elevated. Although no infection was evident, treatment with cefuroxime was initiated.

US-guided 1 cm trocar biopsies were performed twice. They showed dense connective tissue, some macrophages pigmented with foreign material (likely metal), and necrotic debris, suggesting an inflammatory reaction but neither infection nor malignancy (Supplementary Figure S1). After the trocar biopsies, treatment with cefuroxime was replaced with piperacillin–tazobactam. Later on, daptomycin was added to her treatment regimen.

### 2.4. Surgery

Hematologists were confident about the peripheral mechanism for anemia and thrombocytopenia (i.e., pseudotumor-associated bleeding, vasculitis, or another inflammatory reaction). Excision surgery of the suspected pseudotumor was advised and performed to preserve the patient's life.

First, the proximal part of an old anterior scar was incised, and the pelvic part of the suspected pseudotumor was visualized through an iliac window following the medial surface of the ilium. The suspected pseudotumor, along with its capsule, was detached from its margins, requiring it to be opened for safe removal. The femoral nerve and pelvic vessels remained intact. Then, another incision was made to an old posterior scar at the trochanter major. As had been suspected, based on preoperative imaging, the visualized trochanter major was previously fractured. After the capsule was opened and the joint was dislocated, the femoral head part of the endoprosthesis was removed. The femur was lifted anteriorly, and the posterior part of the suspected pseudotumor was detached and removed. Through an anterosuperior opening at the acetabular cup, anteromedial to the ilium, all the remaining suspected parts of the pseudotumor were detached and removed. The acetabular part of the endoprosthesis was found to have partly loosened and was removed, along with the cement and mesh between the bone and cement, without significant bone loss. Surgical drapes were soaked in saline with epinephrin and used for hemostasis of the acetabular region. Then, the remaining femoral part of the endoprosthesis was visualized and removed without fractures. The patient was left with a Girdlestone situation. After additional hemostasis of the acetabular region with TachoSil® Fibrin Sealant patches, hydrogen peroxide lavage was administered, a surgical drain was placed, and the wounds were closed in layers. After collecting bacterial, TB, and fungus samples, all excised tissues were sent for a pathological examination. Macroscopically, the tumor resembled a typical granulation-tissue pseudotumor with no signs of bleeding.

The surgical blood loss of the patient was estimated at 1.9 L. While under general anesthesia, she needed a high-dose infusion of norepinephrine for severe hypotension. Pulmonary bleeding was evident during her intubation. The patient was extubated successfully and transferred back to the intensive care unit. Bleeding from the surgical drain (0.8 L) continued during her first postoperative day but gradually subsided thereafter. The surgical drain was removed on the third postoperative day.

### 2.5. Outcome

Despite the excision of the patient's THA and pseudotumor, as well as RBC and PLT transfusions, her anemia and thrombocytopenia persisted. Immediately after her surgery, her blood lactate levels increased to 9 mmol/L (normal levels: 0.5–2.2 mmol/L) but then normalized within 2 days. Her WBC increased to 45.5 × 10^9^/L and her CRP to 306 mg/L. Despite respiratory support with a high-flow nasal cannula, the patient had objective dyspnea and severe hypoxia due to an alveolar hemorrhage [arterial oxygen tension (PaO_2_): 7.3–8.5 kPa; fraction of inspired oxygen (FiO_2_): 0.4–0.7]. She did not tolerate a non-invasive ventilation mask. She became somnolent at first and then agitated. For sedation, a dexmedetomidine infusion was administered. She experienced a paroxysm of atrial fibrillation. A norepinephrine infusion was administered for hypotension, while furosemide was administered for oliguria. The patient developed moderate kidney insufficiency. Treatment with piperacillin–tazobactam was replaced with meropenem, while daptomycin was continued. She was also administered IVIG and anti-D immunoglobulin.

The histopathological diagnosis of the surgical pseudotumor revealed highly malignant AS. Because of the very poor prognosis of the condition, intensive care was withheld. After the patient was transferred to palliative care, she died within 24 h of severe hypoxia due to alveolar hemorrhage (68 days after her GP had detected her anemia and thrombocytopenia).

**Figure 3:**
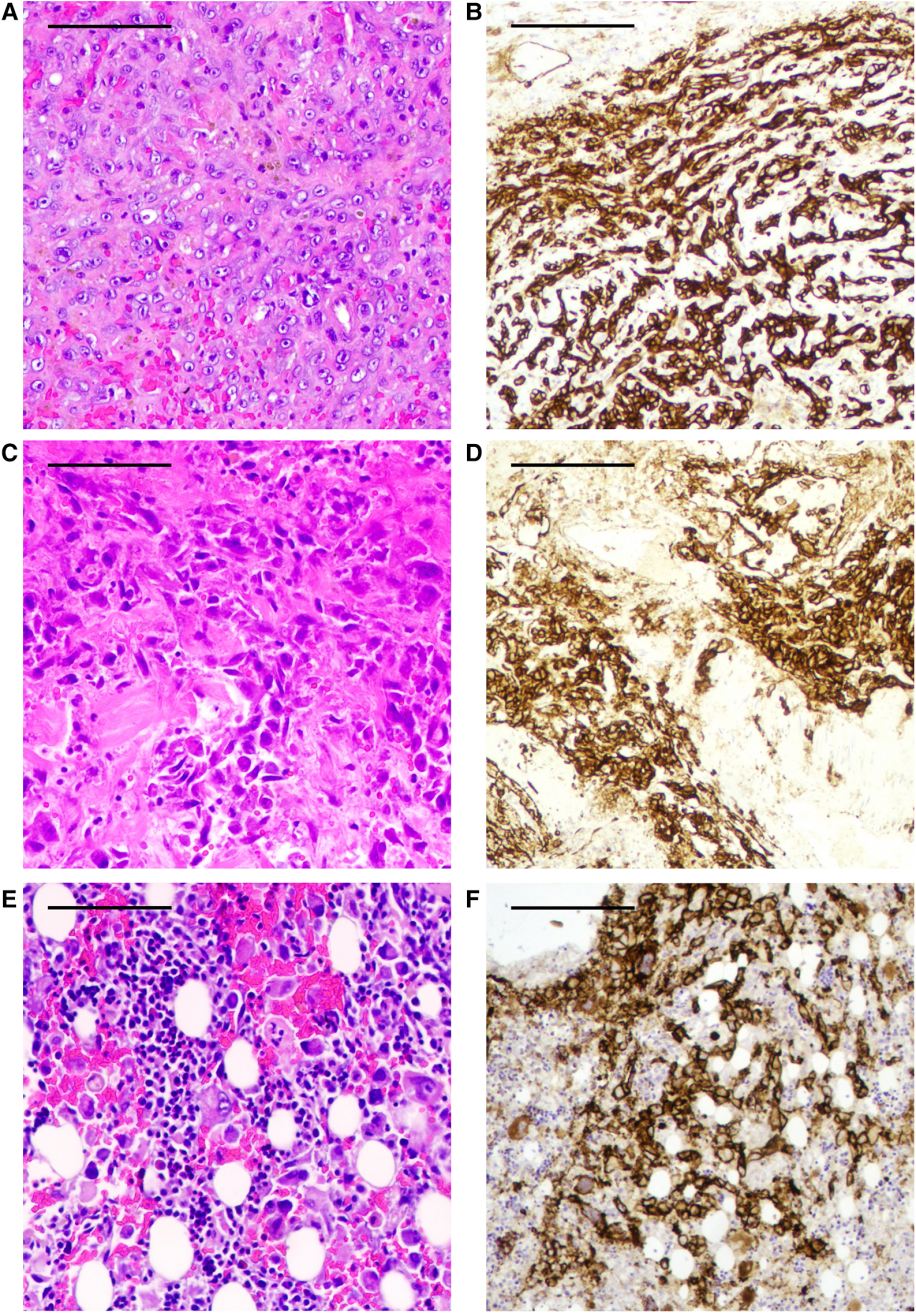
(**A**) Hematoxylin–eosin staining (HE) of autopsy specimens from the THA-associated mass shows a focal, patchy, atypical endothelial cell proliferation. (**B**) The atypical endothelial cell proliferation of the THA-associated mass is highlighted by CD31-positive cells by immunohistochemistry (IHC). (**C**) HE staining of autopsy specimens from the lungs shows atypical endothelial cell proliferation. (**D**) The atypical endothelial cell proliferation of the lungs is shown as CD31-positive cells by IHC. (**E**) HE staining of autopsy specimens from bone marrow shows atypical endothelial cell proliferation. (**F**) The atypical endothelial cell proliferation of the bone marrow is shown as CD31-positive cells by IHC. These findings are diagnostic of angiosarcoma. Scale bar 0.5 mm.

The histopathological diagnosis of her autopsy specimen showed an AS with CD31-positive cells in the pseudotumor (Figures 3A,B), lungs (Figures 3C,D), and bone marrow (Figures 3E,F).

## 3. Discussion

In this case report, we have presented the case of a patient with developmental dysplasia of the hip, a long history of primary and revision MoP THA, leg edema, anemia, and fatal thrombocytopenia. Imaging revealed a soft-tissue mass around the THA, which was presumed to be an inflammatory pseudotumor. Based on the results of various diagnostic tests and her thrombocytopenia's unresponsiveness to PLT transfusions, hematologists suggested a peripheral mechanism of thrombocytopenia (e.g., pseudotumor-associated bleeding, vasculitis, or another inflammatory reaction). Based on the patient's medical history, atypical symptoms, rapid tumor growth, and wide osteolysis, arthroplasty surgeons suspected either a malignancy or an infection. However, histopathological diagnosis using trocar biopsies showed a typical inflammatory pseudotumor. Eventually, the histopathological diagnosis of the surgical resection specimen of the patient revealed highly malignant AS. Postmortem samples showed metastases in the lungs and bone marrow. To our knowledge, this is the first case of AS associated with an orthopedic prosthesis at our high-volume tertiary arthroplasty center.

ASs are an aggressive, extremely rare sarcoma subtype with an approximate incidence rate of two to three new cases in 1,000,000 people per year (25). AS is an infiltrating tumor with a high incidence of recurrence and metastases. At the time of diagnosis, the rate of advanced or metastatic disease varies from 16% to 44%. The 5-year survival rate had been reported as 30%–40%, and overall survival (OS) ranges from 6 to 16 months (26).

ASs are a group of clinically and genetically heterogeneous sarcomas. They may be found in cutaneous lesions (60% of cases, especially in the head and neck region), soft tissues (e.g., the breasts), visceral organs, the retroperitoneum, and bone (26). Although the etiology of AS remains unclear, some risk factors for the condition are known. It can result from secondary to long-term exposure to foreign bodies, including surgical implants (27). Solid-state surface carcinogenesis has been suggested as an etiological mechanism, and it may be associated with the amount and duration of exposure to foreign bodies (28). Other risk factors for secondary angiosarcomas include genetic syndromes, environmental carcinogens and toxins, anabolic steroids, prior radiation, and chronic lymphedema (26, 29). We found 12 reports, including a literature review, on cases of AS among patients with THA (summarized in Supplementary Table S1) (28, 30–35). In these cases, the median time from primary THA to AS diagnosis was 15 years, which suggests that prolonged exposure to THA implants may be associated with AS.

Diagnosing endoprosthesis-related AS is challenging. Biopsies of deeply located tumors remain mostly non-diagnostic (30, 32, 33). In a case series involving five patients, only one AS case was diagnosed using a needle biopsy (30). The histological features of AS vary within and between patients. Typically, AS presents as abnormal, pleomorphic malignant endothelial cells that form sinusoids, multilayered papillary-like projections, and often monocytic infiltration. The cells present endothelial markers, such as vWF, CD31, and Ulex europaeus agglutinin-1 (36).

Imaging plays a key role in screening and differential diagnostics, but it cannot definitively verify the benign or malign nature of a tumor mass or osteolysis (37). A positron emission tomography–CT (PET-CT) scan may provide additional information, especially prior to planning for surgery or other treatments or when detecting metastasis (36, 38). The key to AS diagnostics is the histopathological analysis of a representative biopsy or surgical specimen. In this case report, malignancy was suspected several times, but trocar biopsies suggested a benign pseudotumor. As in many previously reported cases (Supplementary Table S1), AS was only revealed in the histopathological analysis of the patient's surgical specimen.

Clinically, cases of endoprosthesis-associated AS present with a rapidly growing soft-tissue mass, remarkable osteolysis, elevated neutrophils and CRP, fever, and massive bleeding. Among previously reported cases of THA-associated AS (Supplementary Table S1), pain has typically been among the first symptoms (28, 30, 31, 33, 35). Aseptic loosening and remarkable osteolysis were found in almost all cases (30, 32, 34, 35). Leg swelling is common but may also result from DVT (39–41) or the pseudotumor's local compression of the veins (12). Anemia and bleeding were among the main findings in 9 out of 12 cases (28, 30–34). Coagulation dysfunction was not present in two cases despite bleeding problems (31, 32). However, thrombocytopenia was not observed in any previous case report on THA-associated AS.

In 1940, Kasabach and Merritt first reported on thrombocytopenic consumptive coagulopathy associated with a vascular tumor, and it was called the “Kasabach–Merritt phenomenon” (KMP) (42). KMP is mainly associated with kaposiform hemangioendothelioma and tufted angioma among infants and young children. According to the International Society for the Study of Vascular Anomalies classification, thrombocytopenia is not typical of AS (43). Few case reports have presented KMP associated with hepatic AS among adults (44, 45). In KMP, PLTs are activated and trapped in the tumor. The consumption of PLTs, coagulation factors, fibrinolysis, and bleeding may lead to tumor enlargement and progressive pain (46). In addition, transfused PLTs are trapped and destroyed rapidly. Therefore, PLT transfusion is not recommended unless problematic bleeding occurs or the transfusion is needed before invasive surgical procedures. In addition to severe thrombocytopenia, elevated D-dimer, hypofibrinogenemia, and microangiopathic hemolysis may be present (47). In this case report, anemia and bleeding problems were initially moderate, but thrombocytopenia was severe. PLT transfusion did not markedly increase the patient's PLT, as is typical for KMP. Perhaps, as has been previously described for KMP (46), the PLT transfusions contributed to the rapid growth of the pseudotumor.

Due to the rarity of AS, performing prospective randomized controlled trials to compare treatment options is very challenging. Because of a lack of evidence, the optimal treatment of the condition is under debate (26). The first-line treatment is radical surgery if anatomically possible, but positive surgical margins and metastases are common. AS is also responsive to radiotherapy, which is used for inoperable tumors and as adjuvant therapy in addition to surgery. Approximately 50% of AS patients develop recurrence. Chemotherapy (e.g., paclitaxel, doxorubicin, or ifosfamide) may decrease this recurrence rate and is the first-line treatment for metastatic AS. Immunotherapy and targeted therapy may, we hope, improve the OS of AS in the future (26).

AS associated with THA is extremely rare. It was found in approximately 0.1% of THA revision cases in a single-center study (30). Arthroplasty register studies with short-to-medium–term follow-ups may not reveal all AS cases because of the typically very long interval between THA and AS diagnosis. AS may also remain undiagnosed without a thorough histopathologic examination of surgical and postmortem samples. Therefore, the incidence of THA-associated AS may be underestimated. Including information about endoprosthesis-associated malignancies in arthroplasty registries could help estimate the incidence of endoprosthesis-associated AS and evaluate the most effective treatment options for AS.

In this report, we have presented a rare case of THA-associated AS and KMP and the challenges encountered in making a differential diagnosis. Although most periprosthetic pseudotumors are non-malignant, remarkable osteolysis, rapid tumor growth, bleeding, and deterioration in the general wellbeing of a patient may indicate malignancy despite possible benign histology of the biopsies. Due to the unknown etiology and rarity of AS, we can only speculate about the possible causality between THA and AS. Considering the benefits of arthroplasty surgery and the unclear etiology and rarity of AS, we suggest that arthroplasty should not be withheld based on suspicion of an extremely rare occurrence of AS.

## Data Availability

The original contributions presented in the study are included in the article/**Supplementary Material**, further inquiries can be directed to the corresponding author.

## Supplementary material

Supplementary Figure S1 Hematoxylin-eosin staining of a trocar biopsy of the total hip arthroplasty-associated mass shows dense connective tissue, pigmented macrophages (arrows), and necrotic debris, suggesting an inflammatory reaction. Malignancy was not found. Scale bar 1mm.
