# Supplementary material for: Case report and review: Angiosarcoma with thrombocytopenia after total hip arthroplasty
Source: Front Surg. 2023 Jul 31;10:1212491. doi: 10.3389/fsurg.2023.1212491 (PMC10423994; doi:10.3389/fsurg.2023.1212491)
Supplement: Supplementary file 1 [file Table1.pdf]

Supplementary Table 1. Summary of reported cases THA associated AS

Age and sex	Primary symptoms	THAt	Bearing	Diagnostics	Treatment	OS	
75 M	THA, pain for 5 mo, palpable mass in the thigh, anemia	7 y	MoP	CT: osteolysis, needle biopsy (benign)<br>Surgical specimen HPD: epithelioid AS	Hindquarter amputation<br>Chemotherapy	≥ 7 mo	Terrando et al., 2018 (29)
74 M	THA, pain, weight loss, anemia	16 y	MoP	X-ray, CT: osteolysis, aseptic loosening<br>Needle biopsy and intraoperative HPD (benign)<br>Surgical specimen epithelioid AS	Hindquarter amputation<br>Chemotherapy	5 mo	Terrando et al., 2018 (29)
63 F	THA, pain for 1 year	32 y	MoP	X-ray: osteolysis<br>Revision to ceramic-on-ceramic (no AS detected)<br>CT 1 year postoperatively: osteolysis, needle biopsy AS<br>Imaging for staging: lung metastases	Palliative chemotherapy	5 mo	Terrando et al., 2018 (29)
80 M	THA, pain for 5 y<br>Fall, periprosthetic fracture<br>Swelling, anemia	27 y	ND	X-ray: osteolysis; open reduction and internal fixation without revision (no AS detected)<br>CT 7 mo postoperatively: osteolysis, bulky mass<br>Needle biopsy and intraoperative HPD: benign<br>Surgical specimen HPD: epithelioid AS<br>Chest CT: metastases	Hindquarter amputation	4 mo	Terrando et al., 2018 (29)
76 F	THA, gluteal mass	13 y	MoP	Needle biopsy of the gluteal mass (benign)<br>Surgical specimen HPD of mass resection: epithelioid AS<br>CT lung metastases	Re-excision, chemotherapy	9 mo	Terrando et al., 2018 (29)
ND	THA after giant cell tumor of bone<br>Pain, fluctuating mass	14 y	MoP	Clinical exam: fluid collection<br>CT, MRI: tissue mass, fluid collection<br>Revision surgery HPD: benign<br>Recurrent fluid, arteria rupture, DVT<br>Biopsy (vena cava filter surgery) HPD: angiosarcoma	Chemotherapy withdrawn	5–6 mo	Zhu et al., 2016 (30)
64 F	THA, developmental dysplasia of the hip<br>Fall, periprosthetic fracture<br>anemia, hematoma	≥ 30 y	MoP	CT + MRI: wide osteolysis, hematoma<br>CT: Pleural effusion, cytology (not malignant)<br>Hematoma evacuation, surgical HPD: benign<br>Hemipelvectomy surgical samples HPD: angiosarcoma	Hemipelvectomy with colostomy	ND	Sánchez-García et al., 2019 (31)
69 F	THA due to osteoarthritis<br>Muscle detachment, revision<br>Aseptic loosening, second revision<br>Skin purpura, hemorrhage	8 mo	ND	Second skin biopsy HPD: angiosarcoma<br>CT, PET-CT: large masses in the pelvic region, no metastases	Radiotherapy	40 days	Lee et al., 2021 (32)

84 F	THA<br>Aseptic loosening, revision twice<br>Pain, deterioration of mobility<br>Purple nodules and erythema	30 y	MoP/<br>CoP/<br>MoP	X-ray: osteolysis<br>Two earlier revisions: no HPD samples taken<br>HPD of skin lesion biopsy: angiosarcoma	Palliative radiotherapy	ND	Mallick et al., 2009 (33)
78 M	ND	17 y	ND	MRI: tissue mass and osteolysis<br>Revision surgery samples HPD: epithelioid AS	Palliative care	D	Agaimy et al., 2016 (34)
55 M	ND	8 y	ND	CT: tissue mass and osteolysis<br>Revision surgery samples HPD: epithelioid AS<br>Lung metastases	Surgery, radiotherapy, chemotherapy	≥ 17 mo	Agaimy et al., 2016 (34)
72 F	THA, developmental dysplasia of the hip<br>Pain, swelling, malaise	12 y	MoP	X-ray: severe osteolysis<br>Revision surgery, no HPD<br>Open biopsy of tumor tissue HPD: epithelioid hemangioendothelioma (i.e., angiosarcoma)	ND	ND	van der List et al., 1988 (35)
74 F	THA, developmental dysplasia<br>Revision for aseptic loosening, limb edema, pain, bruises	34 y	MoP	CT + MARS-MRI: large mass, osteolysis<br>Two trocar biopsies HPD: pseudotumor (benign)<br>Surgical resection samples and autopsy HPD: angiosarcoma, metastases in lungs, bone marrow	Palliative care	68 days	The case described in the current report

Abbreviations: AS (angiosarcoma); CoP (ceramic-on-polyethylene); CT (computed tomography); D (died shortly after diagnosis); F (female); HPD (histopathological diagnosis); M (male); MARS-MRI (metal-artifact-reducing sequence magnetic resonance imaging); mo (months); MoP (metal-on-polyethylene); MRI (magnetic resonance imaging); ND (not described), OS (overall survival time), PET-CT (positron emission tomography–computed tomography); THA (total hip arthroplasty); THAt (time from primary THA to examination); and y (years).
