# Supplementary figures and images for: Case report and review: Angiosarcoma with thrombocytopenia after total hip arthroplasty
Source: Front Surg. 2023 Jul 31;10:1212491. doi: 10.3389/fsurg.2023.1212491 (PMC10423994; doi:10.3389/fsurg.2023.1212491)

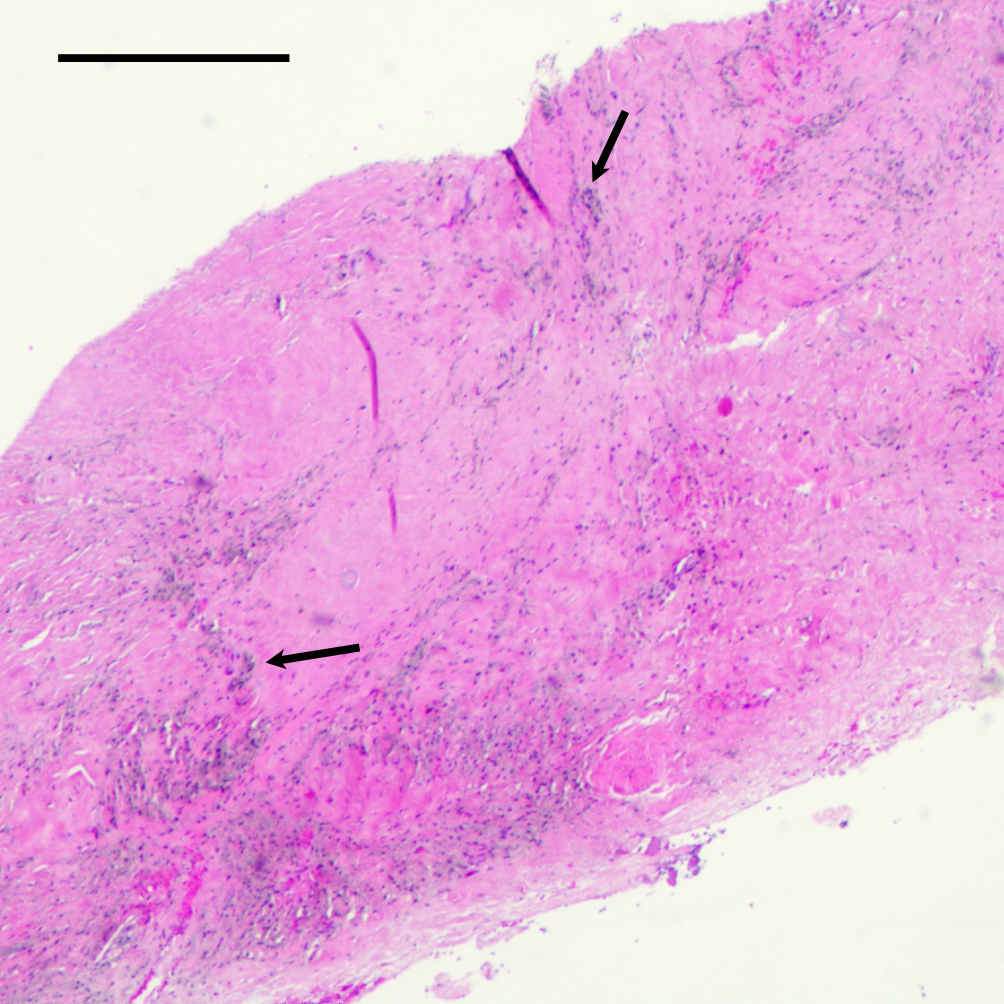

Supplement: Supplementary Figure S1 — Hematoxylin-eosin staining of a trocar biopsy of the total hip arthroplasty-associated mass shows dense connective tissue, pigmented macrophages (arrows), and necrotic debris, suggesting an inflammatory reaction. Malignancy was not found. Scale bar 1mm. [file Image1.tif]
